# Application of metagenomic shotgun sequencing to detect vector-borne pathogens in clinical blood samples

**DOI:** 10.1371/journal.pone.0222915

**Published:** 2019-10-02

**Authors:** Prakhar Vijayvargiya, Patricio R. Jeraldo, Matthew J. Thoendel, Kerryl E. Greenwood-Quaintance, Zerelda Esquer Garrigos, M. Rizwan Sohail, Nicholas Chia, Bobbi S. Pritt, Robin Patel

**Affiliations:** 1 Division of Infectious Diseases, Mayo Clinic, Rochester, Minnesota, United States of America; 2 Department of Surgery, Mayo Clinic, Rochester, Minnesota, United States of America; 3 Center for Individualized Medicine, Mayo Clinic, Rochester, Minnesota, United States of America; 4 Division of Clinical Microbiology, Mayo Clinic, Rochester, Minnesota, United States of America; Johns Hopkins University, UNITED STATES

## Abstract

**Background:**

Vector-borne pathogens are a significant public health concern worldwide. Infections with these pathogens, some of which are emerging, are likely under-recognized due to the lack of widely-available laboratory tests. There is an urgent need for further advancement in diagnostic modalities to detect new and known vector-borne pathogens. We evaluated the utility of metagenomic shotgun sequencing (MGS) as a pathogen agnostic approach for detecting vector-borne pathogens from human blood samples.

**Methods:**

Residual whole blood samples from patients with known infection with *Babesia microti*, *Borrelia hermsii*, *Plasmodium falciparum*, *Mansonella perstans*, *Anaplasma phagocytophilum* or *Ehrlichia chaffeensis* were studied. Samples underwent DNA extraction, removal of human DNA, whole genome amplification, and paired-end library preparation, followed by sequencing on Illumina HiSeq 2500. Bioinformatic analysis was performed using the Livermore Metagenomics Analysis Toolkit (LMAT), Metagenomic Phylogenetic Analysis (MetaPhlAn2), Genomic Origin Through Taxonomic CHAllenge (GOTTCHA) and Kraken 2.

**Results:**

Eight samples were included in the study (2 samples each for *P*. *falciparum* and *A*. *phagocytophilum*). An average of 27.5 million read pairs was generated per sample (range, 18.3–38.8 million) prior to removal of human reads. At least one of the analytic tools was able to detect four of six organisms at the genus level, and the organism present in five of eight specimens at the species level. *Mansonella* and *Ehrlichia* species were not detected by any of the tools; however, mitochondrial cytochrome c oxidase subunit I amino acid sequence analysis suggested the presence of *M*. *perstans* genetic material.

**Conclusions:**

MGS is a promising tool with the potential to evolve as a non-hypothesis driven diagnostic test to detect vector-borne pathogens, including protozoa and helminths.

## Introduction

Vector-borne pathogens constitute a significant public health concern, comprising more than 17% of infections globally and causing an estimated 700,000 deaths annually [1]. They are also a major contributor to emerging infections, with nine new mosquito- or tick-borne diseases having been introduced or discovered in the United States since 2004 [2]. Infections with these emerging pathogens are likely under-recognized due to the lack of widely-available laboratory tests. There is an urgent need for advancement in diagnostic modalities to detect new and known vector-borne pathogens.

Metagenomic shotgun sequencing (MGS) is a pathogen agnostic approach with the potential to revolutionize microbial diagnostics, as it allows detection of diverse microorganisms and associated virulence and antimicrobial resistance genes, as well as discovery of novel pathogens. The utility of MGS in clinical practice, and details surrounding its ideal performance, are being established. Compared to single gene amplification approaches, such as 16S rRNA gene PCR/sequencing, which can only detect a specific group of organisms, MGS provides the possibility of detecting bacteria, viruses, fungi, protozoa and helminths in a single test. Instead of clinical suspicion- or hypothesis-driven testing which relies on competency and experience of the healthcare provider, a clinical test utilizing MGS can allow unbiased detection of microorganisms. This capability has been evaluated in clinical studies to detect pathogens from a variety of clinical specimens, including blood (whole blood, buffy coat, serum, and plasma), urine, stool, respiratory secretions, and synovial and spinal fluid [3–8]. Most of these studies have focused on detection of bacteria, viruses or fungi, but not protozoa or helminths. For the true potential of MGS as an inclusive pathogen agnostic test to be realized, it should be optimized to detect all potential prokaryotic and eukaryotic pathogens, including those that are fastidious or non-culturable.

A challenge in achieving this goal is the development and optimization of sample preparation methods that effectively decrease the amount of host nucleic acids, lyse host cells to release intracellular organisms and lyse microbial cells to release nucleic acids, without negatively affecting the quantity of targeted nucleic acid in the sample. Another hurdle is effectually parsing and analyzing the millions of sequenced reads to identify known pathogens. The success of accurately identifying detected sequences is limited by the presence of a matching sequence in the database of the analytic tool(s) being applied, and therefore, it is important to select the ideal tool(s) to use. Studies comparing the results of different metagenomic pipelines for vector-borne pathogens are lacking in the literature.

To evaluate the potential of MGS to detect vector-borne organisms in blood, we studied specimens containing three eukaryotic pathogens (*Babesia microti*, *Plasmodium falciparum*, and *Mansonella perstans*), two rickettsiales (*Ehrlichia chaffeensis* and *Anaplasma phagocytophilum*) and one spirochete (*Borrelia hermsii*). Out of these, four are intracellular pathogens (*Babesia*, *Plasmodium*, *Ehrlichia* and *Anaplasma* species) and two extracellular pathogens (*M*. *perstans* and *B*. *hermsii*). We analyzed the sequenced nucleic acids from these samples using four analytical tools–Livermore Metagenomics Analysis Toolkit (LMAT), Metagenomic Phylogenetic Analysis (MetaPhlAn2), Genomic Origin Through Taxonomic CHAllenge (GOTTCHA) and Kraken 2.

## Methods

This study was approved by Mayo Clinic Institutional Review Boards (ID: 16–005221). The Institutional Review Board waived the requirement to obtain consent.

### Specimens

Residual whole blood samples (0.5–1 ml), collected and stored in EDTA tubes from patients with known infection with *B*. *microti* (BM), *B*. *hermsii* (BH), *P*. *falciparum* (PF-1 and PF-2), *M*. *perstans* (MP), *A*. *phagocytophilum* (AP-1 and AP-2), and *E*. *chaffeensis* (EC) were studied. The original diagnoses were made using conventional microscopy and/or PCR-based methods. *M*. *perstans* was identified using Knott’s concentration, with *P*. *falciparum* identified using thin blood films. The remaining organisms were identified using laboratory-developed, clinically-validated PCR assays [9, 10], with *B*. *hermsii* identification confirmed by Sanger sequencing of a fragment of *glpQ* following PCR amplification. All samples had been stored at 4°C, with nucleic acid extracted within 4 days of collection, except for the *M*. *perstans* sample which had been frozen at -80°C for 13 years.

### Sample preparation

Sample preparation was performed in a laminar flow hood. The MolYsis Complete5 kit (Molzym, Bremen, Germany) was used for DNA extraction and removal of host DNA. Due to the low sample volume, the “small size sample protocol” was used per the manufacturer’s recommendation, with the exception that elution was performed with 70 μl of deionized water. TE buffer and *Corynebacterium glutamicum* (CG) ATCC 13032 suspended in TE buffer (10^5^ CFU/ml) were extracted alongside the test specimens as external negative and positive controls (NC and PC-CG), respectively.

Whole genome amplification was performed using the REPLI-g Single Cell Kit (Qiagen, Hilden, Germany) in a separate room from sample preparation. Amplified DNA was purified using Agencourt AMPure XP beads (Beckman Coulter, Brea, CA) with bead volume of 1.5X sample volume. Purified DNA was diluted to a concentration of 2 ng/μl by adding molecular grade water (DNase/RNase free). A final 50 μl sample volume was submitted for sequencing.

### Sequencing

Library preparation and massive parallel sequencing were performed in a physically separate location by the Clinical Genome Sequencing Laboratory, Division of Biochemical Genetics, Department of Laboratory Medicine and Pathology (Mayo Clinic Rochester, MN). The samples were sheared to 550 base pairs (bp) using a LE220 Sonicator (Covaris, Woburn, MA). Paired-end libraries were prepared with the TruSeq Nano library preparation kit (Illumina, San Diego, CA) on a BioMek FX liquid handling station (Beckman Coulter, Brea, CA) followed by sequencing on HiSeq 2500 in rapid run mode (Illumina) resulting in paired reads of 150 bp in length.

### Bioinformatics analysis

Raw reads from the sequencer were inspected for residual adapters using Atropos v1.1.19 [11]. Putative human reads were removed using BioBloom Tools v2.1.1 [12] prior to analysis by GOTTCHA, LMAT and MetaPhlAn2. Low-complexity reads were masked from analysis using VSEARCH v2.8.4 [13]. The following tools were used for taxonomy calling: LMAT v1.2.6 [14] using database kML+H.noprune.4-14.2025, MetaPhlAn2 v2.7.0 [15], GOTTCHA v2.1 beta with databases based on RefSeq release 75 [16], and Kraken2 v2.0.7beta [17] with bacterial, archaeal, fungal, viral and protozoan databases created in October 2018. An average of 27.5 million read pairs was generated per sample (range, 18.3–38.8 million) prior to removal of human reads. Statistical analysis of results generated between the different databases was not performed because of small sample size.

### Publication of sequences

Human reads were removed, and the raw sequence data for each sample were deposited in the Sequence Read Archive (SRA) database at the National Center for Biotechnology Information (NCBI) under BioProject accession number PRJNA518922.

## Results

### Analysis at the genus level

Comparison of the four tools vis-à-vis genus-level identification is shown in Table 1. Interpretation from GOTTCHA was split into bacteria and protozoa as different datasets were applied for the two analyses. Four of six pathogens (*Babesia*, *Borrelia*, *Anaplasma* and *Plasmodium* species) were detected by at least one of the analytic tools at the genus level. However, a difference was noted in sensitivity of detection between the different analytic tools. For PF-1, 92,711 reads were attributed to *Plasmodium* by Kraken 2 but only 17,849 reads by GOTTCHA-Protozoa. Similarly, for PF-2, higher reads matches were reported by Kraken 2 than LMAT or GOTTCHA. *Babesia* had over 2.5M and 1.5M reads assigned by GOTTCHA-Protozoa and Kraken 2, respectively, but only 733 reads assigned by LMAT. Neither *Mansonella* nor *Ehrlichia* was detected by any of the tools.

**Table 1:** Analysis at the genus level.

Specimen	GOTTCHA-Bacteria		GOTTCHA-Protozoa		LMAT	MetaPhlAn2	Kraken 2
	Read count	Relative abundance*	Read count	Relative abundance*	Read count	Relative abundance*	Reads
**BH**	5,397,402	99.7	0	0	5,155,036	99.989	6,908,083
**PF-1**	0	0	17,848	72.6	73,803	73.245	92,711
**BM**	0	0	2,191,962	100	733	0	1,588,363
**MP**	0	0	0	0	0	0	0
**PF-2**	0	0	12,772	100	55,402	0	176,571
**EC**	0	0	0	0	0	0	0
**AP-1**	2,542	12.7	0	0	1,812	77.060	1,890
**AP-2**	5	11	0	0	5	0	4
**PC-CG**	19,833,473	99.5	0	0	36,980,606	99.997	38,562,714

*Relative abundance is expressed as percentage. Relative abundance estimated by GOTTCHA was read-based and used rolled up depth of coverage for calculation. MetaPhlAn2 provided organismal relative abundance (in terms of number of cells rather than fraction of reads). Relative abundance by GOTTCHA and MetaPhlAn2 are not directly comparable. BH, *B*. *hermsii*; PF, *P*. *falciparum;* BM, *B*. *microti;* MP, *M*. *perstans;* AP, *A*. *phagocytophilum;* EC, *E*. *chaffeensis;* PC-CG, Positive control—*C*. *glutamicum*.

The read score for the second *Anaplasma* sample was low, likely because of a low load of microorganism in the sample (crossing point >40 cycles by real-time PCR). Of the two *Plasmodium* samples, one had higher read scores and read counts compared to the other. MetaPhlAn2 was able to detect only one of the *Plasmodium* samples, PF-1, which had higher read counts than PF-2 by other tools. All four tools detected the positive control. The negative control had reads that matched *C*. *glutamicum*, suggesting cross-contamination between positive and negative controls.

### Analysis at the species level

The species-level analysis is shown in Table 2. Most reads that matched to the pathogens at genus levels also matched at species level with few exceptions. *B*. *microti* was not identified by LMAT at the species level, with the 733 reads assigned to the genus *Babesia* split between *B*. *equi* (472) and *B*. *bovis* (217). There was a lower level of confidence at the species level for *P*. *falciparum*. For sample PF-2, out of 171,946 reads at the *Plasmodium* genus level, only 29,981 corresponded to *P*. *falciparum*, with more reads assigned to *P*. *vivax* (38,302) than *P*. *falciparum*.

**Table 2:** Analysis at the species level.

Specimen	GOTTCHA-Bacteria		GOTTCHA-Protozoa		LMAT	MetaPhlAn2	Kraken 2
	Read count	Relative abundance*	Read count	Relative abundance*	Read count	Relative abundance*	Reads
**BH**	5,393,026	71	0	0	4,997,666	99.977	6,885,897
**PF-1**	0	0	17,230	46.0	72,490	73.245	79,934
**BM**	0	0	2,191,962	100	0	0	1,570,492
**MP**	0	0	0	0	0	0	0
**PF-2**	0	0	12,334	37.4	35,874	0	29,647
**EC**	0	0	0	0	0	0	0
**AP-1**	2,542	12.7	0	0	1,810	77.060	1,876
**AP-2**	5	12.4	0	0	5	0	4
**PC-CG**	19,832,203	98.7	0	0	29,169,927	99.997	29,349,222

*Relative abundance is expressed as percentage. Relative abundance estimated by GOTTCHA was read-based and used rolled up depth of coverage for calculation. MetaPhlAn2 provided organismal relative abundance (in terms of number of cells rather than fraction of reads). Relative abundance by GOTTCHA and MetaPhlAn2 are not directly comparable. BH, *B*. *hermsii*; PF, *P*. *falciparum;* BM, *B*. *microti;* MP, *M*. *perstans;* AP, *A*. *phagocytophilum;* EC, *E*. *chaffeensis;* PC-CG, Positive control with *C*. *glutamicum*.

Complete details of the results from all four analytic tools are available as supporting information (S1 Table).

### Sankey interpretation

**Fig 1:**
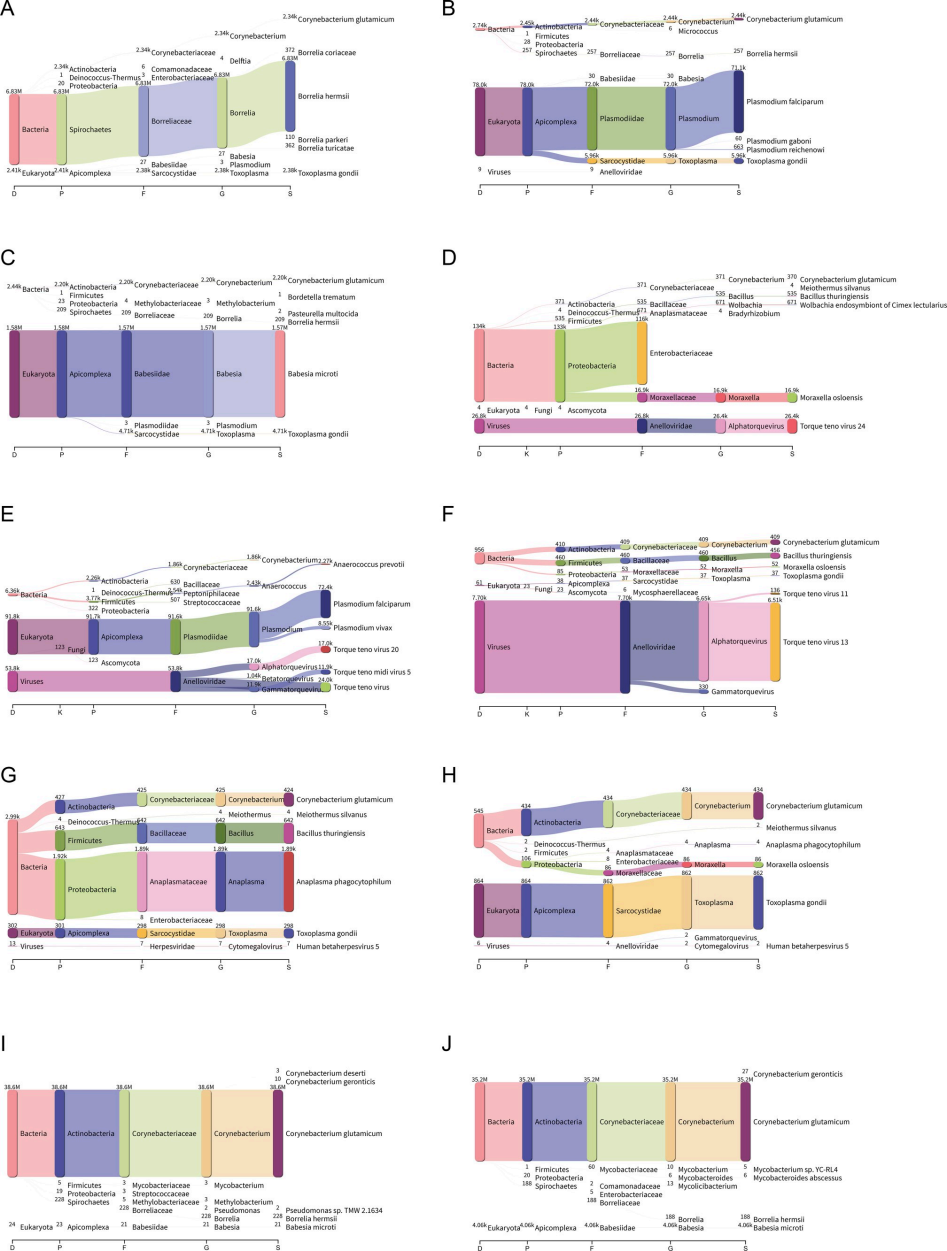
Sankey diagrams of Kraken 2 report from eight clinical samples and two controls. A) *B*. *hermsii*, B) *P*. *falciparum* (PF-1), C) *B*. *microti*, D) *M*. *perstans*, E) *P*. *falciparum* (PF-2), F) *E*. *chaffeensis*, G) *A*. *phagocytophilum* (AP-1), H) *A*. *phagocytophilum* (AP-2), I) Positive control–*C*. *glutamicum*, J) Negative control.

Pavian is an online interactive browser that provides a visual report from Kraken, MetaPhlAn2 and Centrifuge [18, 19]. Sankey diagrams obtained from the Kraken 2 reports from the eight samples and two controls are as shown in Fig 1.

### Identification by mitochondrial gene sequence matching

The filarial worm *M*. *perstans* was missed by all analytic tools studied. However, the presence of filarial DNA was suggested by reads assigned to other nematodes. GOTTCHA invertebrates matched 16,873 reads to phylum Nematoda and 16,871 reads to order Spirurida. Based on k-mers, LMAT assigned 3,295 reads to genus *Brugia* and 2,173 reads to *Onchocerca*. *Wolbachia* species, a Gram-negative bacterium which can be an endosymbiont of filarial worms, including *M*. *perstans*, also generated 10,967 and 1,941 reads with LMAT and Kraken 2, respectively [20]. None of the other samples had reads originating from a nematode or *Wolbachia* species.

The genome of *M*. *perstans* has not been sequenced as of January 2019 which likely explains the inability of these analytic tools to accurately identify the organism. There is, however, a protein sequence of the *M*. *perstans* mitochondrial cytochrome c oxidase subunit I (Cox1) gene publicly available (UniProt accession A0A1M4NFV5). This sequence was matched against the sequence data obtained from the sample that was positive for *M*. *perstans* by PCR. Using DIAMOND v0.9.22 [21], we searched the raw reads against the *M*. *perstans* Cox1 amino acid sequence. We also included Cox1 sequences for *B*. *microti* (accession A0A0K3API2) and *P*. *falciparum* (accession Q02766). Results expressed as reads per kilobase per million (RPKM) mapped from this analysis are shown in Table 3. 574,807 RPKM belonged to *M*. *perstans* Cox1, with four other samples having low levels of RPKM for *M*. *perstans* Cox1 (6–24), likely as a result of cross-contamination. Similarly, the cytochrome c oxidase subunit I gene for *Babesia* and *Plasmodium* species were matched against all the samples and generated 1,146, 3,426 and 163,400 RPKMs of the relevant organisms for BM, PF-1 and PF-2, respectively, with 31 *P*. *falciparum* Cox1 RPKMs noted in the *M*. *perstans* sample.

**Table 3:** Abundance of Cox1 amino acid sequences for *M*. *perstans*, *B*. *microti* and *P*. *falciparum* against all the samples with counts normalized to RPKM.

	Gene Family		
Blood Sample	*Mansonella perstans* Cox1	*Babesia microti* Cox1	*Plasmodium falciparum* Cox1
*B*. *hermsii*	0	0	0
*P*. *falciparum* -1	0	0	3,426
*B*. *microti*	0	1,146	0
*M*. *perstans*	574,807	0	31
*P*. *falciparum* -2	24	0	163,400
*E*. *chaffeensis*	6	0	0
*A*. *phagocytophilum* -1	12	0	0
*A*. *phagocytophilum* -2	6	0	0

## Discussion

Our study findings suggest that MGS can successfully detect a wide variety of vector-borne human pathogens, including protozoa and one helminth. We also report novel strategies to optimize MGS results for detecting eukaryotic organisms in cases where sufficient genomic sequencing information is not available. These observations support the evolving role of MGS as a potential pathogen agnostic clinical test where diagnostic evaluation is not hampered by clinical suspicion for a particular infection by the ordering clinician.

Because of the large size of the sequenced data, MGS dictates the use of computational tools to analyze the fragmented sequences, measure the length of the covered genome and provide a taxonomical assignment. Previously, NCBI BLAST was used to perform this task but as the size of the sequencing dataset increased over time, other tools that are faster and more efficient than BLAST were developed. Many of these tools are publically available for use. Broadly, these tools can be divided in those that use all available sequences (e.g., GOTTCHA [16], LMAT [14], Kraken [17, 22]) and those that utilize a set of marker genes (e.g., MetaPhlAn2 [15]). GOTTCHA is a gene-independent and signature-based method which employs a database of unique 24 base pair (bp) fragments for generating a taxonomic profile. In contrast, LMAT and Kraken rely on exact k-mer (sequence fragments of length “k”) matches, instead of alignments. Kraken uses a default k of 31 whereas LMAT has a default k of 20. This difference in k-mer length is significant since in general, long k-mers may be more specific and short k-mers more sensitive. Also, Kraken assigns the matched k-mer to the lowest common ancestor (LCA), while LMAT stores the source genome instead of LCA. Therefore k-mers assigned to a particular taxon may vary depending on the tool used. Kraken 2 is a newer version of Kraken (default k of 35) with faster database build times, smaller database sizes, and faster classification speeds. MetaPhlAn2 uses clade-specific marker genes for taxonomical profiling. Since only genes are assigned, the process is faster than mapping reads to an entire genome [23]. The unique characteristics of these computational tools make it necessary to use multiple methods when analyzing samples for uncommon organisms.

As we demonstrate in this study, organisms that have whole genome sequences available from multiple strains are likely to be picked up by most analytic tools, whereas uncommon microbes that are yet to be fully sequenced render analysis more challenging. At the species level, GOTTCHA, LMAT, MetaPhlAn2 and Kraken 2 were able to detect 5, 4 (missed *B*. *microti*), 3 (missed *B*. *microti*, *P*. *falciparum*-2) and 5 organisms out of the 8 samples, respectively. Notable is the discrepancy between the reads attributed to *P*. *falciparum* by Kraken 2 (n = 92,711) *versus* 17,849 read by GOTTCHA-Protozoa. GOTTCHA, which is alignment based, generally returns fewer hits than kmer based tools like Kraken 2. *M*. *perstans*, *E*. *chaffeensis* and one sample with *A*. *phagocytophilum* were missed by all four tools. Besides lack of sequence data in the databases (the *M*. *perstans* sample), other reasons why organisms were missed could be low load of the pathogen (the *A*. *phagocytophilum* sample had a crossing point of >40 cycles and the *E*. *chaffeensis* sample had a crossing point of 35 cycles), loss of DNA as a result of prolonged storage (the *M*. *perstans* sample) or loss of DNA during processing (enrichment and/or extraction).

Although *M*. *perstans* was not detected by any of the analytic tools initially, analysis of the cytochrome c oxidase subunit I amino acid sequence suggested that the genetic material of the filarial worm was in fact present in the final analyzed sequence but was not detected due to the absence of *M*. *perstans* genomic sequence in the databases utilized. To further test this hypothesis, we queried the cytochrome c oxidase sequences of *Babesia* and *Plasmodium* species against all our samples and found that we were able to match sequences to the specimens harboring these species, with little cross-detection (Table 3). Targeting mitochondrial genes is a potential method to detect eukaryotic organisms in cases where insufficient whole genomic sequence information is available.

Depending on the enrichment method used, eukaryotic pathogen nucleic acid may be inadvertently removed from the sample, alongside host DNA, prior to sequencing. In this study, we used MolYsis^™^ Complete5 for removal of host DNA as well as extraction of microbial nucleic acid. This step utilizes a chaotropic buffer that selectively lyses human/animal cells followed by degradation of the free nucleic acid by chaotrope-resistant MolDNase B. This chaotropic buffer could potentially lyse a eukaryotic pathogen in the sample along with human/animal cells, leading to loss of the eukaryotic pathogen’s nucleic acid. An alternative might be the use of NEBNext® microbiome DNA enrichment kit which selectively binds and removes the CpG-methylated host DNA. However, a head to head comparison of MolYsis^™^ and NEBNext® suggested that NEBNext® does not enrich bacterial DNA to the extent that MolYsis^™^ does [24]. Others have evaluated the nonionic detergent saponin for selective lysis of human cells and release intracellular bacteria for improved detection [25, 26]. However, saponin may also lead to unbiased lysis of eukaryotic cells, including pathogen cells. Cell-free DNA sequencing has been used successfully to detect pathogens from plasma and thereby eliminate the need for host cell lysis [27]. On the other hand, this approach may theoretically miss intracellular pathogens. Bioinformatics tools may be employed to improve pathogen to human read ratios by removing reads matched to human k-mers. We used BioBloom Tools v2.1.1 to filter out human and PhiX sequences prior to analysis by LMAT, GOTTCHA and MetaPhlan2. However, bioinformatic tools can also potentially remove reads from pathogens. Alternatively, sequencing may be performed without biochemical and bioinformatic enrichment but this requires deeper sequencing which increases cost. Further research is warranted to develop and optimize a sample preparation approach to improve universal detection of intracellular and eukaryotic pathogens of different types.

The primary limitation of this study is its small sample size, which precluded statistical analysis. To more fully understand the limitations of the methods and analytic tools studied, a larger study, adding a focus on pathogens with unknown or draft genome sequences, including intracellular and eukaryotic organisms, is needed. In addition, we only studied whole blood and not other blood components (plasma, serum, buffy coat). Also, we did not sequence RNA, which would be needed to identify RNA viruses. Finally, we found evidence of cross-contamination, a challenge especially germane to clinical implementation.

That each sample sequenced with MGS generated a number of reads attributable to multiple organisms would obviously render it challenging to differentiate between true pathogens and contaminants were this approach to be used clinically; contaminants may derive from reagents or cross-contamination between samples at any one (or more) of multiple steps. Therefore, if this approach is to be applied clinically, it will be critically important to incorporate methods to prevent and detect cross-contamination so as not to issue false positive results. Cross-contamination may be minimized by performance of individual steps in separate, purpose-build facilities used solely for MGS; utilization of microfluidics or automated sample preparation methods may help abrogate contamination. Clinical utilization will require MGS-specific quality assurance and quality control process, including incorporation of both internal and external controls. Internal controls should be included in amounts that do not interfere with detection of varying amounts of diverse pathogens in patient samples, all the while maximizing their ability to detect interfering substances in individual samples and address cross-contamination, ideally by incorporating unique internal controls into each patient sample. Use of artificial sequences can potentially fulfill the need for unique internal controls that would not interfere with pathogen interpretation [28, 29]. Likewise, external positive controls should consist of mixtures of organisms unlikely to be observed in humans, but able to quality control the process for detection of diverse organism-types while simultaneously revealing any cross-contamination present. Negative controls also help address cross-contamination—in the current study, we detected *C*. *glutamicum* in the negative control -; negative controls can also help define background deriving from reagents used, which may vary over time and should therefore be tracked within and between runs. Potential solutions for index mis-assignment from multiplexing, another source of false-positive results, include use of the Free Adapter Blocking Reagent and of Unique Dual Indexes (Illumina).

For clinical application of MGS, rigorous interpretive thresholds will need to be established for interpretation of sequence data, based, for example, on read counts, relative abundance, read scores, depths of coverage and results of sequencing of external controls and clinical samples (alongside their internal controls) on the same run and historically [4, 8]. To the extent that it is likely to be impossible to mitigate all background microbial reads, clinical application of MGS may be limited in its ability to detect pathogens present at low abundance.

In conclusion, MGS is a promising tool with the potential to evolve as a non-hypothesis driven diagnostic test. However, further work needs to be done to optimize sample preparation methods and expand the reach of analytic tools, especially for vector-borne pathogens, including protozoa and helminths. The absence of whole genome sequence of *M*. *perstans* in publically-available databases limits the power of MGS for detection of this organism, a situation that likely applies to other organisms. Efforts should be made to generate good quality whole genome sequence data for poorly-represented organisms, such as *M*. *perstans*.

## Supporting information

S1 Table Analysis of data by all four databases. (XLSX)
